# Multi-class subarachnoid hemorrhage severity prediction: addressing challenges in predicting rare outcomes

**DOI:** 10.1007/s10143-025-03678-9

**Published:** 2025-07-10

**Authors:** Muhammad Mohsin Khan, Adiba Tabassum Chowdhury, Md. Shaheenur Islam Sumon, Shaikh Nissaruddin Maheboob, Arshad Ali, Abdul Nasser Thabet, Ghaya Al-Rumaihi, Sirajeddin Belkhair, Ghanem AlSulaiti, Ali Ayyad, Noman Shah, Anwarul Hasan, Shona Pedersen, Muhammad E. H. Chowdhury

**Affiliations:** 1 Neurosurgery Department, Hamad Medical Corporation, Doha, Qatar; 2 Department of Electrical and Electronics Engineering, University of Dhaka, Dhaka, Bangladesh; 3 Department of Electrical Engineering, Qatar University, Doha, 2713 Qatar; 4 Department of Surgical Intensive Care Unit, Hamad Medical Corporation, Doha, Qatar; 5 Department of Industrial and Mechanical Engineering, Qatar University, Doha, Qatar; 6 Medical Science College of Medicine, Qatar University, Doha, Qatar

**Keywords:** Subarachnoid hemorrhage, Severity prediction, Modified rankin scale, Imbalanced data, Electronic health record

## Abstract

Accurately predicting the severity of subarachnoid hemorrhage (SAH) is critical for informing clinical decisions and improving patient outcomes. This study addresses the challenges of imbalanced data in SAH severity classification by employing the Modified Rankin Scale (MRS) within a three-stage classification framework. We utilize a three-stage approach to effectively categorize SAH severity. In the first stage, we performed binary classification, grouping SAH severity into “Good Outcome” (class 0), which includes MRS levels 0, 1, 2, and 3, and “Poor Outcome” (class 1), encompassing levels 4, 5, and 6. Feature selection was done using a Random Forest algorithm to identify the top 20 features for the SAH severity prediction. We evaluated thirteen machine learning models at each stage, selecting the top-performing classifiers to optimize results. The dataset comprised 535 samples across seven MRS severity levels and was validated using 5-fold cross-validation and diverse subgroups to ensure robust model performance across various scenarios. Binary classification in the first stage achieved approximately 90% accuracy with Extra Trees. In the second stage, targeting the “Good Outcome” group, the Random Forest model reached 88% accuracy, while in the third stage, it achieved 86% accuracy for the “Poor Outcome” group. By increasing accuracy across unbalanced classes and emphasizing its potential for practical use, the multi-stage technique presents a promising solution for predicting the severity of SAH. Future research will concentrate on additional tuning to improve the model’s efficacy in actual healthcare environments.

**Supplementary Information:**

The online version contains supplementary material available at 10.1007/s10143-025-03678-9.

## Clinical trial number

Not applicable.

## Introduction

**Fig. 1:**
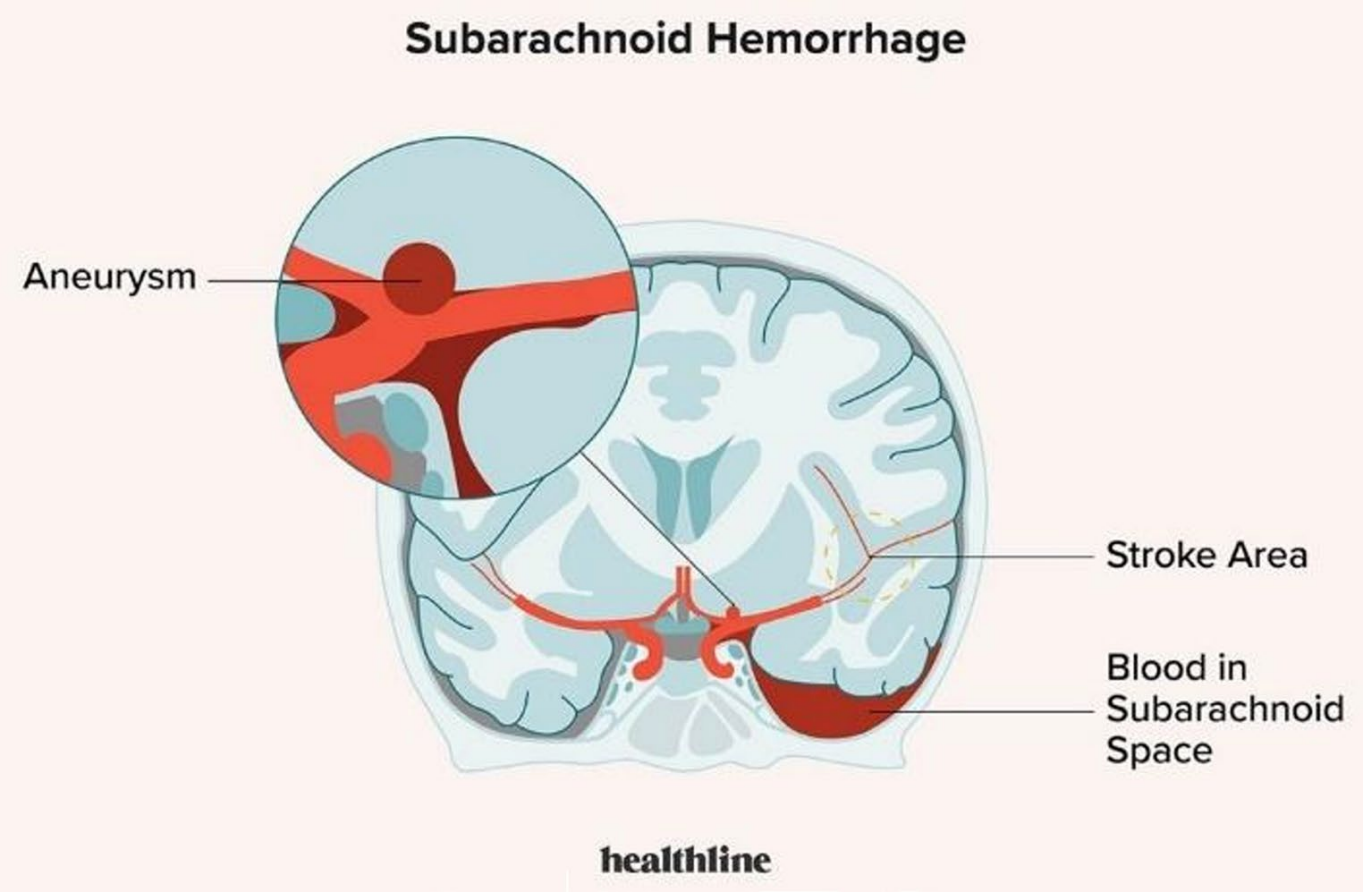
Visual Representation of Subarachnoid Hemorrhage [3]

A dreadful neurological emergency of a subgroup of strokes known as subarachnoid hemorrhage (SAH) is characterized by blood seeping into the subarachnoid space (Fig. 1), primarily as a result of cerebral aneurysm rupture. Its symptoms, which are frequently sudden and severe, include severe headaches, nausea, altered awareness, and neurological impairments [1]. Although trauma or arteriovenous malformations (AVMs) can sometimes induce SAH, cerebral aneurysm rupture is the primary cause. Because of its high morbidity and death rates, early detection is essential [2].

Cerebral aneurysm rupture is the main cause of SAH, which can result in subarachnoid bleeding, a sharp rise in intracranial pressure, brain compression, ischemia, and potentially lethal perfusion deficiencies brought on by vasospasm [4]. The clinical presentation of SAH is remarkably dramatic, with patients frequently reporting sudden, intense headaches along with symptoms like photophobia, nausea, stiff neck, and neurological impairments [5]. With an incidence of 10 per 100,000 per year, SAH makes up 5% of all strokes and primarily affects women between the ages of 40 and 60. Risk factors for SAH include smoking, excessive alcohol use, high blood pressure, and a family history of brain aneurysms [6, 7]. Head CT is the first line of treatment for SAH because it can detect blood in the subarachnoid space. If CT is not conclusive, lumbar puncture is used as a follow-up because xanthochromia in the cerebrospinal fluid validates the diagnosis [8]. Healthcare professionals must have a thorough understanding of SAH’s origins, symptoms, and diagnostic procedures because it is a serious neurological emergency that needs to be identified and treated quickly to lower morbidity and death [9].

In order to maximize results and direct individualized clinical care, it is crucial to predict the severity of SAH as it allows clinicians to tailor care plans, select appropriate treatments, and quickly address high-risk cases to improve outcomes [10]. Prognostic insights from SAH severity prediction allow for well-informed decision-making, reasonable expectations for recovery, and fruitful dialogue between patients, families, and doctors [11]. By anticipating the severity of SAH, proactive therapy can be provided to avoid complications. For high-risk patients, this includes focused monitoring, blood pressure management, nimodipine, and other early therapies that lower the risk of seizures, ischemia, vasospasm, and hydrocephalus [12]. By identifying patients in need of intensive care, allowing targeted therapy for high-risk patients, and preventing needless interventions for low-risk instances, SAH severity prediction facilitates effective resource allocation [13]. SAH severity prediction models support research and quality improvement by identifying risk factors, refining treatment protocols, and personalizing care, thereby enhancing outcomes and guiding clinical decisions for this critical condition [14, 15].

The unequal distribution of severity classes among patients [16, 17] and complicated nature [18] with varied character [19, 20] of SAH are the main causes of challenges faced during SAH severity prediction generally. One of the difficulties we encountered with our dataset collected from Hamad General Hospital for this study was the unequal representation of SAH severity classes; uncommon outcomes were underrepresented, which could have skewed the model in favor of majority classes. Model training is impacted, and real-world generalizability is limited by the lack of data for severe SAH cases. Furthermore, it was difficult to obtain strong validation across several patient groups, particularly when dealing with rare and unbalanced outcome data. To tackle these multi-class prediction issues for SAH severity categorization, our objective of this research was to -.

- This study aims to predict SAH severity using a three-stage classification approach with the Modified Rankin Scale (MRS) to address challenges in imbalanced data.
- A custom SAH dataset of 535 samples was developed, with the top 20 predictive features selected using Random Forest, and multiple machine learning models evaluated across three classification stages.
- The model achieved 90% accuracy in initial binary classification, followed by 88% accuracy in the “Good Outcome” group and 86% accuracy in the “Poor Outcome” group, demonstrating effective performance on imbalanced classes.
- This multi-stage classification framework shows strong potential for clinical application in SAH severity prediction, with future improvements planned for further optimization in real-world settings.

## Literature review

Researchers are using machine learning techniques more often to create prediction models for SAH severity categorization in an effort to overcome these constraints. Duan et al. (2016) [21] investigated variables linked to poor clinical outcomes in elderly patients receiving endovascular therapy (EVT) for aneurysmal subarachnoid hemorrhage (aSAH) using factors like conventional prognostic scoring methods, such the Fisher grade and the Hunt and Hess grading scale, in the field of predicting outcomes for aSAH. The crucial significance of aneurysmal SAH, which has a fatality rate above 30%, is highlighted by Petridis et al. (2017) [22]. The authors highlight the great sensitivity and specificity of computed tomography (CT) in identifying blood in the basal cisterns within the first 12 h following SAH. Hostettler et al. (2018) [23] used decision tree analysis based on clinical and laboratory data to develop prediction models for outcome parameters in aSAH patients. Jaja et al. (2018) [24] advanced the field by creating useful prediction tools integrating clinical and neuroimaging data, improving prognostic accuracy for functional outcomes and mortality. Ramos et al. (2019) [25] acknowledged the limitations of traditional logistic regression models in predicting delayed cerebral ischemia (DCI) in aSAH patients. Early bedside prediction models for clinical outcomes following aSAH were identified as necessary by van Donkelaar et al. (2019) [26]. Xia et al. (2020) [27] addressed this need by applying random forest machine learning techniques and showed superior predictive performance for adverse outcomes in patients with anterior communicating artery (ACoA) aneurysms. The difficulties in forecasting worse functional outcomes in patients with aSAH were brought to light by Eagles et al. (2020) [28], who emphasized the significance of determining both modifiable and non-modifiable variables. In the meanwhile, Dengler et al. (2021) [29] demonstrated how machine learning techniques may help get around the drawbacks of conventional scoring systems and enhance outcome prediction for patients with aSAH. By examining outcome factors in SAH patients, Rava et al. (2021) [30] provided insight into how endovascular therapy (ET) affects patient outcomes. Wang et al. (2022) [31] made noteworthy progress by creating an XGBoost-based prognostic model with improved predictive value for mortality and functional outcomes in patients with aSAH, despite the conflicting results on the impact of ET on outcomes. To estimate the 28-day mortality risk in patients with SAH who are not traumatized or who have had trauma, Miao et al. (2023) [32] created predictive nomogram models. For SAH patients, the nomograms provided better predictive accuracy compared to widely used grading methods. Studies on outcomes and complications predicted in SAH patients were evaluated by Salman et al. (2023) [33]. With the inclusion of new categories, the Modified Rankin Scale (MRS) as an instrument of severity broadens the scope of the Rankin Scale and offers a more sophisticated evaluation of functional outcomes, especially for stroke patients [34, 35]. While Burth et al. (2023) [36] examined outcome factors for patients suffering from SAH, including the impact of endovascular therapy (ET), they also pointed out that the results were not entirely definitive on how ET affected patient outcomes. Finding risk factors for complications and unfavorable outcomes in aSAH patients with initially good neurological state was acknowledged by Lenkeit et al. (2024) [37].

In order to balance the class distribution, oversampling procedures Synthetic Minority Over-sampling Technique (SMOTE) and Random Oversampling produce synthetic samples to increase the minority class and reduce class imbalance was used in Dengler et al. (2021) [29]. Algorithms like Cost-Sensitive Decision Trees and Cost-Sensitive Support Vector Machines can enhance model performance on unbalanced datasets by strongly punishing misclassifications of the minority class [38]. Resampling strategies or loss function modifications to account for class imbalance can be added to techniques like Bagging, Boosting, and Random Forests to manage unbalanced data [39]. By interpolating between already-existing instances in the feature space, augmentation techniques like SMOTE and ADASYN produce synthetic data points, boosting the diversity of the minority class [40]. Deep learning models known as Generative Adversarial Networks (GANs) are made up of a discriminator network that has been trained adversarially and a generator network. For unbalanced datasets, GANs may be trained to produce realistic synthetic samples that closely mimic the minority class distribution, hence enhancing the training set [41, 42]. SMOTE oversampling and undersampling are used in techniques like SMOTE combined with Edited Nearest Neighbors (SMOTEENN) and synthetic minority oversampling technique + Tomek Link (SMOTETomek) to eliminate noisy or borderline instances from the majority class and create new examples for the minority class [43, 44].

Medical prediction tasks have been transformed by machine learning approaches, which provide strong instruments for deciphering intricate healthcare data and enhancing clinical decision-making [45]. Shi et al. (2020) used CNNs to assist in prognostic evaluation by automatically learning hierarchical features from image data and identifying minor patterns indicative of SAH severity [46]. Time-series data and EHRs have been used to predict clinical outcomes in SAH patients, and LSTM and RNN models have been used to model temporal trends in patient data because of their superiority at processing sequential data [47, 48]. Techniques like Random Forests, Gradient Boosting Machines (GBMs), and AdaBoost efficiently capture complicated correlations and lower the chance of overfitting in SAH severity prediction tasks [49]. Clinicians may trust and evaluate predictive models for SAH severity prediction due to XAI techniques like feature significance analysis, SHapley Additive exPlanations (SHAP), and LIME (Local Interpretable Model-agnostic Explanations), which improve model interpretability [50, 51].

## Methodology

**Fig. 2:**
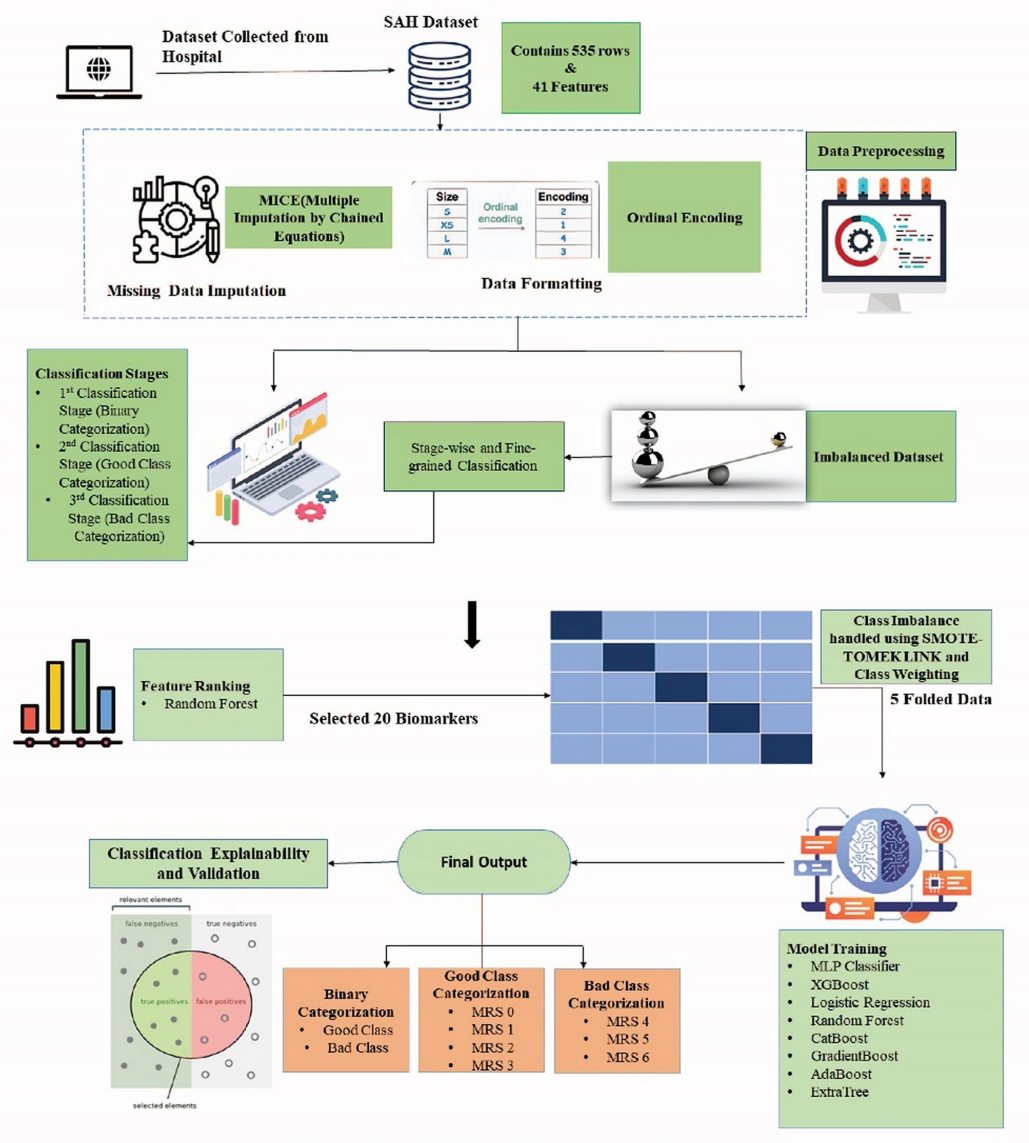
Summary of the methodology adopted for severity prediction of SAH

This section outlines the methods we used to predict SAH severity in our study. Our suggested strategy aims to close the gap between clinical decision-making in SAH care and predictive analytics by using a multi-stage approach. The methodology of this study for predicting the severity of subarachnoid hemorrhage (SAH) is comprehensively illustrated in Fig. 2.

Data collection, feature screening, and ground truth definition initiate the procedure. Data preprocessing, which encompasses ordinal encoding, is implemented to standardize the input data. To improve the model’s performance, the imbalance in class distribution is then rectified. The most significant features are chosen through feature ranking, and they are subsequently validated using 5-fold cross-validation to guarantee their robustness. The classification is performed in three phases, with each stage increasingly refining the classification within subgroups. Ultimately, the model’s performance is assessed, resulting in the provision of final predictions and pertinent evaluation metrics to verify its efficacy.

### Dataset description

The data for the study was obtained from the electronic medical records of Hamad General Hospital. Anonymized patient data were collected from the neurosurgery and critical care units of a tertiary care hospital using a retrospective study with the IRB approval from the local IRB team (Protocol# MRC-01-24-031). The inclusion criteria encompassed patients aged 18 years and older with subarachnoid hemorrhage confirmed through neuroimaging, with severity classified using the World Federation of Neurological Surgeons scale and the modified Fisher grading. Cases with incomplete data, concurrent traumatic brain injuries, or conditions unrelated to subarachnoid hemorrhage were excluded. Data extraction involved structured queries in the hospital’s electronic medical records, gathering demographic, clinical, radiological, and laboratory information, as well as outcomes like mortality and vasospasm.

Our dataset includes 535 patients’ electronic health record data, which includes a wide range of clinical and demographic characteristics relevant to the prediction of SAH severity. The dataset has rows that correspond to individual patient records. These rows have many columns that capture different parts of the patient’s medical history, demographics, test findings and clinical markers. Among them, we used the MRS as the ground truth severity class. Each score, which ranges from 0 to 6, corresponds to a distinct degree of functional impairment as shown in Table 1 [52].

**Table 1:** Modified Rankin scale-wise physical symptoms

Scores	Symptoms
Score 0	Absolutely no symptoms.
Score 1	No major impairment. Capable of doing all daily tasks, notwithstanding some symptoms.
Score 2	Mild impairment. Capable of handling personal matters on their own without help, but unable to complete all prior tasks.
Score 3	Moderate disability. Able to walk without assistance but need some aid.
Score 4	Severe disability. Incapable of taking care of one’s own basic requirements without aid and unable to walk without help.
Score 5	Extremely disabled. Needs round-the-clock nursing care; bedridden, incontinent.
Score 6	No longer alive.

The MRS offers a comprehensive measure of overall functional impairment, capturing the broader impact of SAH on patients’ daily activities and quality of life. This contrasts with other severity assessment scores like the Hunt and Hess Score or Fisher Score, which concentrate primarily on specific aspects of SAH severity (e.g., severity of headache, presence of intraventricular hemorrhage) [52].

**Fig. 3:**
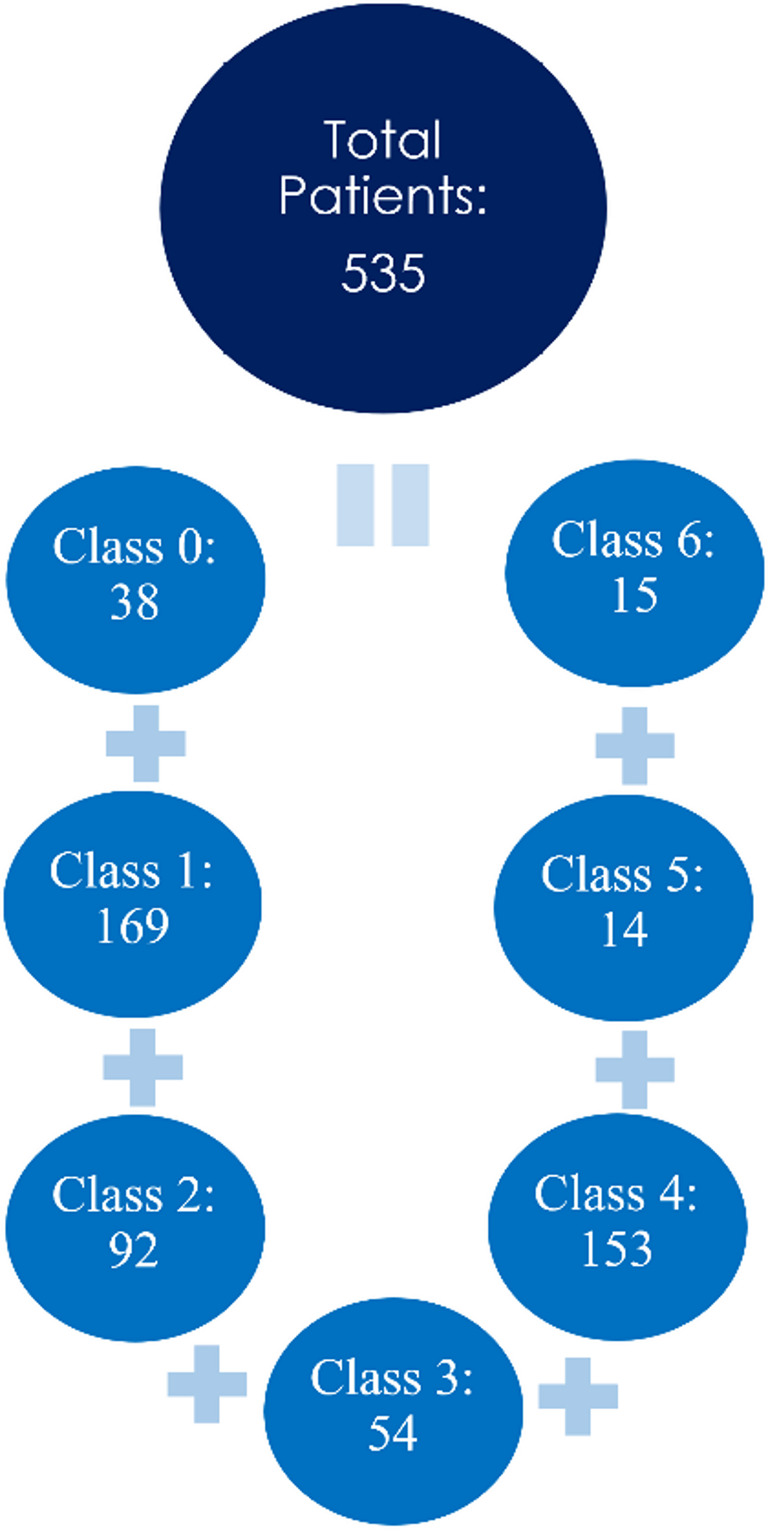
Severity class-wise (modified rankin scale) number of patients in the dataset

The distribution of severity classes in our dataset is Fig. 3.

It is noteworthy that a large imbalance is revealed by the distribution of data, especially for classes 5 and 6, which correspond to the highest degrees of impairment. The small number of cases in both classes—14 and 15 individuals, respectively—reflects the difficulties in gathering information about patients who have the greatest degrees of impairment. Because these patients are frequently very sick, they may not be able to have a full diagnostic evaluation or be admitted to the hospital, which underrepresents them in clinical databases.

### Dataset preprocessing and splitting

Demographics (e.g., “Gender,” “DOB”), medical history (“Hypertension”, “CAD”, “Migraines”), lifestyle factors (“Smoking”), and clinical indicators (“Glassgow Coma Scale (GCS)”, “blood pressure”, “Hunt and Hess Score (H&H Score)”) are among the variables that are included in our dataset. A detailed statistical analysis of some prime demographics and biomarkers of the patients are given in Supplementary Table S1.

We translated categorical variables into comprehensible numerical values using ordinal encoding [53], where bigger values correspond to more severe traits and lower values to less severe ones. In the cases of “Never Smoked,” “Former Smoker,” and “Current Smoker,” for example, “Smoking” was encoded as 0, 1, and 2. Technical validity and interpretability for clinical insights were ensured by using imputation to manage missing results and a multi-stage classification strategy to predict SAH severity.

We use a 5-fold cross-validation process to guarantee comprehensive model evaluation, which enhances the prediction models’ generalizability, robustness, and dependability. 80% of the patients are included in the training set, while the remaining 20% are included in the test set.

### Handling imbalanced classes

A major difficulty in our dataset is the uneven distribution of severity classes, especially when it comes to how the most severe stroke level (classes 5 and 6) in the MRS are represented. It’s important to note that data imbalance posed serious difficulties for us, especially for classes 5 and 6. To rectify this imbalance, we had to use a range of methods and strategies. We used a multi-stage classification method to resolve this imbalance in order to lessen its negative effects on model training and prediction performance.

#### Stage-wise classification

To address the imbalance in severity classes, we used a stage-wise classification approach. We divided classes 0, 1, 2, and 3 into one “Good Outcome” (Class 0) and classes 4, 5, and 6 into one “Bad Outcome” class (Class 1). Through the consolidation of less severe classes into one category and more severe classes into another, this binary classification strategy assisted in achieving a more balanced distribution of severity classes.

#### Fine-grained classification

Following the initial stage’s identification of the “Good Outcome” and “Bad Outcome” classifications, we carried out fine-grained classification within each category. We divided the severity levels into classes 0, 1, 2, and 3 specifically for the “Good Outcome " class (Class 0), which made it possible to analyze severity in this area in greater detail. In a similar vein, we separately classified levels 4, 5, and 6 for the “Bad Outcome” class (Class 1) for further prediction.

#### Class weighting

To give minority classes greater weights and majority classes lower weights during model training, we used class weighting for which model gives minority classes greater consideration during training, class weighting helps to lessen the impact of class imbalance on prediction performance [54].

#### SMOTE

We used SMOTE to rectify class imbalance during training and enhance the resilience of the model. SMOTE is a data augmentation technique created to address the problem of imbalanced datasets, in which the target variable has a non-uniform distribution. One class, the minority class, is noticeably underrepresented in these datasets in comparison to the majority class [29].

### Feature ranking

In order to prevent overfitting, which occurs when a model overfits to the complexities of the training data and reduces its accuracy when applied to fresh datasets, feature ranking is essential. The importance of feature selection is especially evident given that our dataset has 37 features. In order to help reduce the complexity of the model, features are ranked according to their relevance levels. This method reduces the chance of overfitting by improving the model’s capacity for generalization through the deliberate selection of key characteristics. We employed the Random Forest feature ranking technique in this investigation.

### Performance evaluation metrics

The evaluation of the model performance cannot be done using accuracy alone [55–57]. To ensure the reliability of the results, a diverse set of assessment criteria was employed, recognizing that relying solely on accuracy was insufficient. Several metrics can be discerned through the application of the following formulas, ranging from Eq. 1 to 5:

1 $$\:Accuracy\:\left(A\right)=\:\frac{TP+TN}{TP+TN+FP+FN}$$

2 $$\:Precision\:\left(P\right)=\frac{TP}{TP+FP}$$

3 $$\:Recall\:\left(R\right)=\frac{TP}{TP+FN}$$

4 $$\:Specificity\:\left(S\right)=\frac{TN}{TN+FP}$$

5 $$\:F1-Score\:\left(F1\right)=2\frac{Precision\times\:Recall}{Precision+Recall}$$

where TP, TN, FP, and FN refer to True Positive, True Negative, False Positive, and False Negative, respectively. The ROC (Receiver Operating Characteristic), AUC (area under the curve), and confusion matrix can be used to assess the classification performance and to obtain valuable insights about the model performance.

## Results

The multi-class prediction model’s performance for SAH severity prediction is provided in this section. The performance measures for each severity class, including accuracy, precision, recall, specificity, F1-score, and AUC, are presented to evaluate the model’s efficacy across the entire range of SAH severity. The analysis compares the model against baseline models, highlighting improvements achieved by the proposed methodology. Detailed model predictions are examined, addressing both strengths and weaknesses. Special attention is given to the worst SAH classes (Class 5 and 6), offering insights into the model’s performance in predicting critical cases. In this section, we also discuss the top features used by the model, followed by the results based on these top features. The evaluations aim to provide a comprehensive understanding of the model’s predictive performance and its clinical implications.

### Feature selection

The predictive accuracy of our model for SAH severity is substantially improved through comprehensive feature engineering and rigorous feature selection. Utilizing Random Forest, XGBoost, and Extra Trees, we meticulously identify the most informative variables, with Random Forest ultimately selected for the initial binary classification due to its superior performance in distinguishing “good” from “poor” SAH outcomes. For further refined classification within the “Good” and “Bad” categories, iterative application of ensemble techniques identifies features predictive of specific severity levels, ensuring the model’s interpretability and generalizability across SAH categories. Correlation analysis mitigates multicollinearity, thereby enhancing prediction reliability while preprocessing steps such as missing data imputation, ordinal encoding, and normalization establishing a standardized feature scale. Informed by domain knowledge, our approach integrates critical prognostic indicators, even in limited-data contexts, to provide accurate and insightful predictions on SAH severity.

**Fig. 4:**
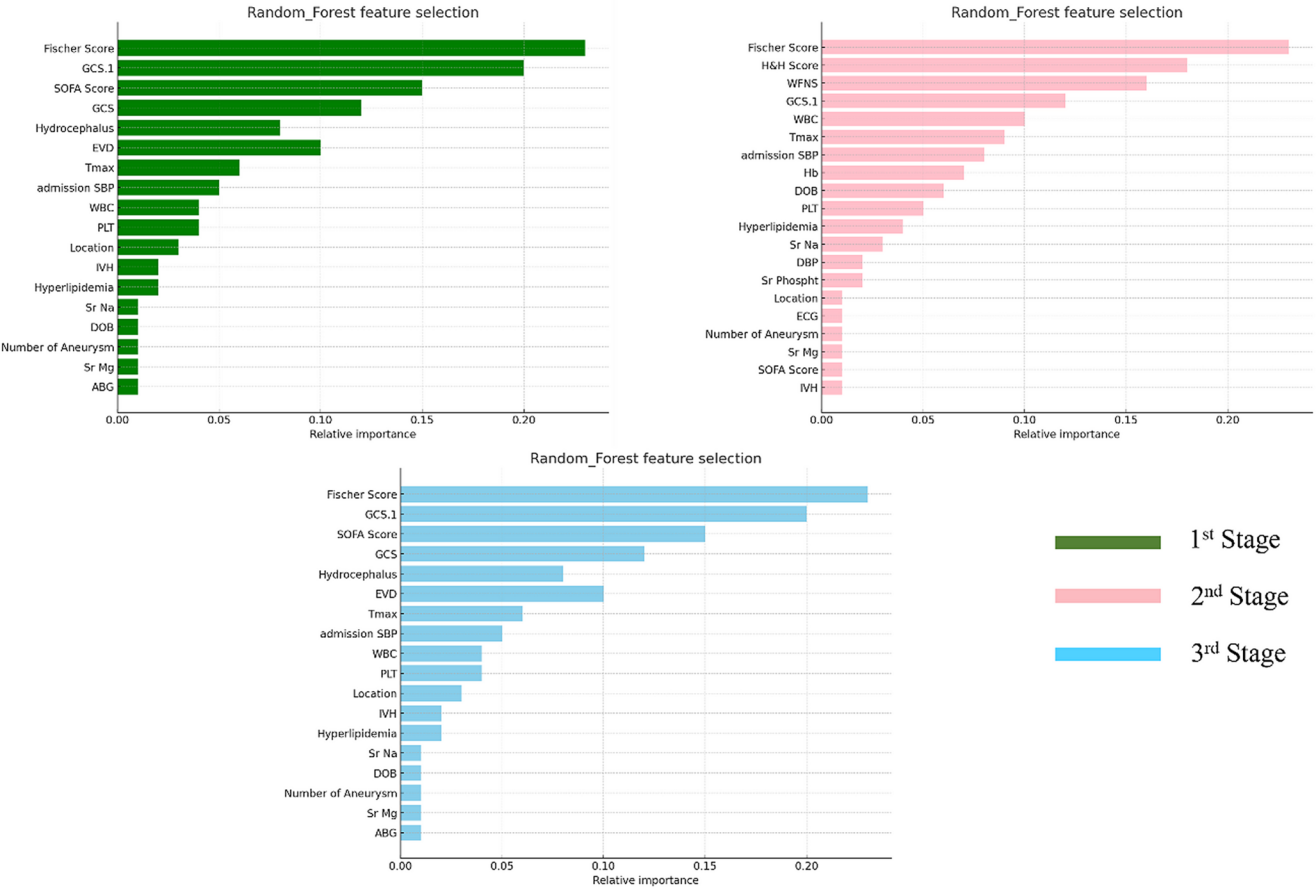
Top 20 features selection of all the three classification stages using Random Forest feature selection algorithm

Figure 4 illustrates the relative importance of features determined by the Random Forest model across three stages of classification: first stage (A), second stage (B), and third stage (C). The features are ranked according to their contribution to the model’s performance in each stage. The Fisher Score, GCS, Sequential Organ Failure Assessment (SOFA) Score, and H&H Score are notable features that consistently demonstrate high importance. Some features, such as admission Systolic Blood Pressure (SBP), White Blood Count (WBC), and Platelet Count (PLT), maintain relevance throughout, while the distribution of feature importance varies across stages. These insights offer a thorough comprehension of feature prioritization to ensure precise classification throughout the stages.

### Machine learning model performances

The performance of our multi-class SAH severity prediction model varies among severity classes. A variety of performance indicators, such as accuracy, precision, recall, specificity, F1-score, and area under the curve (AUC), are used to assess the efficacy of the model. First Stage Classification (0, 1), Detailed Categorization - Good Outcome (classes 0, 1, 2, 3), and Detailed Categorization - Poor Outcome (classes 4, 5, 6) are the three groups based on severity classes that comprise the analysis.

#### First classification stage

In the first classification stage, we performed binary classification. The model demonstrates strong performance across classifiers in the binary classification task, effectively distinguishing between two severity groups. Table 2 highlights the performance of several machine learning models based on key evaluation metrics, including accuracy, precision, recall, specificity, F1-score, and AUC. ExtraTrees exhibited superior overall performance, achieving the greatest accuracy (90.09%), F1-score (90.19%), and AUC (96.04%). Additional significant performers are Logistic Regression (88.78% accuracy, AUC: 95.11%), and ElasticNet (88.97% accuracy, AUC: 95.13%). Conversely, models such as SVM (77.75% accuracy, AUC: 87.12%) and GradientBoosting (85.60% accuracy, AUC: 91.98%) had relatively inferior performance. This overview emphasizes the significance of optimizing and selecting the appropriate algorithm according to dataset attributes.

**Table 2:** Top-performing ML models for first classification stage

Models	Accuracy (%)	Precision (%)	Recall (%)	Specificity(%)	F1-score (%)	AUC (%)	
MLP	87.85	88.34	87.85	87.61	87.98	93.92	
LDA	87.85	89.37	87.85	90.54	88.09	94.5	
XGB	85.98	87.22	85.98	87.44	86.23	95.51	
Random Forest	87.66	88.52	87.66	88.58	87.84	94.71	
Logistic Regression	88.78	89.62	88.78	89.95	88.95	95.11	
SVM	77.75	80.16	77.75	79.48	78.25	87.12	
**ExtraTrees**	**90.09**	**90.48**	**90.09**	**90.10**	**90.19**	**96.04**	
AdaBoost	87.10	87.85	87.10	87.49	87.28	93.47	
KNeighbors	86.91	87.89	86.91	87.93	87.12	92.26	
GradientBoosting	85.60	86.47	85.60	85.92	85.81	91.98	
CatBoost	86.35	87.59	86.35	87.90	86.59	94.85	
LGBM	85.98	87.22	85.98	87.44	86.23	90.79	
ElasticNet	88.97	89.76	88.97	90.05	89.13	95.13	

The AUC curve and confusion matrix are provided in Supplementary Figs. 1 S and 2 S, respectively. The efficacy of a binary classification model is assessed by this confusion matrix. The model demonstrated exceptional performance in the identification of both classes, accurately predicting 311 true negatives (88.10%) and 168 true positives (92.31%). Nevertheless, it incorrectly identified 42 instances as false positives (11.90%) and 14 instances as false negatives (7.69%).

#### Second classification stage

In the second classification stage, detailed categorization of SAH severity into good outcome classes (classes 1, 2, 3, and 4) demonstrates the competitive performance of our model across several classifiers, as shown in Table 3.

**Table 3:** Top-performing ML models for the second classification stage

Models	Accuracy (%)	Precision (%)	Recall (%)	Specificity (%)	F1-score (%)	AUC (%)
MLP	85.83	85.78	85.83	91.43	85.60	90.21
LDA	77.62	80.06	77.62	90.93	77.76	89.03
XGB	85.83	85.90	85.83	91.63	85.53	90.05
**Random Forest**	**87.25**	**87.27**	**87.25**	**91.79**	**87.06**	**90.78**
Logistic Regression	84.70	84.96	84.70	91.42	84.29	89.05
SVM	86.11	86.26	86.11	91.73	85.82	88.1
ExtraTrees	85.26	85.26	85.26	91.43	84.98	90.52
AdaBoost	59.20	54.20	59.20	64.94	47.20	66.02
KNeighbors	74.22	74.78	74.22	80.31	72.33	86.45
GradientBoosting	86.68	86.71	86.68	91.63	86.49	89.34
CatBoost	86.68	86.71	86.68	91.63	86.49	90.29
LGBM	86.11	86.26	86.11	91.73	85.82	90.31
ElasticNet	84.70	84.96	84.70	91.42	84.29	89.05

The Random Forest Classifier achieved an accuracy of 87.54%, demonstrating strong precision, recall, F1-score, and ROC curve, as shown in Supplementary Fig. 3S, along with a detailed confusion matrix depicted in Supplementary Fig. 4S. This indicates the model’s ability to accurately identify cases within the less severe SAH categories. However, the confusion matrix reveals that over 20% of class 2 patients were misclassified as class 1, likely due to similarities in clinical presentation between the two classes. These shared features include modest motor impairments, moderate neurological deficits, and mild cognitive deficits. Misclassification may also arise from variability in symptom presentation, individual patient characteristics, and nuances in clinical assessment.

#### Third classification stage

In the third classification stage, the model encountered challenges in classifying poor outcome severity classes (classes 4, 5, and 6), as depicted in Table 4. These challenges primarily stem from unbalanced data and insufficient support for some classes. Despite these difficulties, the Random Forest Classifier outperformed other models, achieving an accuracy of 86.26%. However, it is important to note that even the Random Forest Classifier demonstrated relatively low performance in terms of accuracy, recall, F1-score, ROC curve, and confusion matrix, as shown in Fig. 10. This highlights the inherent difficulty of accurately predicting cases within severe SAH categories. The confusion matrix further reveals that approximately 86% of class 5 patients and 53% of class 6 patients were misclassified as class 4 patients, likely due to data limitations that adversely impacted the training process.

**Table 4:** Top-performing ML models for the third classification stage

Models	Accuracy (%)	Precision (%)	Recall (%)	Specificity (%)	F1-score (%)	AUC (%)	
MLP	69.78022	73.339419	69.78022	82.924786	71.487163	66.36	
LDA	63.186813	80.519259	63.186813	79.561525	69.701299	69.67	
XGB	76.923077	81.257599	76.923077	89.539603	78.7671	79.26	
**Random Forest**	**86.263736**	**82.28022**	**86.263736**	**92.722728**	**83.234564**	**79.51**	
Logistic Regression	68.681319	77.096588	68.681319	81.98983	72.39107	75.35	
SVM	69.78022	76.871113	69.78022	80.480824	72.921008	73	
ExtraTrees	81.868132	77.352647	81.868132	90.514968	79.410357	78.23	
AdaBoost	71.428571	74.962028	71.428571	83.993576	73.124053	58.49	
KNeighbors	47.252747	78.928535	47.252747	69.831587	55.719759	62.12	
GradientBoosting	81.318681	79.860391	81.318681	89.1498	80.378276	74.78	
CatBoost	82.967033	80.467033	82.967033	90.21859	81.624907	79.3	
LGBM	75.274725	77.475607	75.274725	87.756403	76.219315	71.49	
ElasticNet	67.582418	77.279163	67.582418	82.135843	71.762115	74.76	

SHAP (SHapley Additive exPlanations) [58] analysis is a frequently used method in model interpretability to analyze the contribution of specific features in machine learning models. In the classification of subarachnoid hemorrhage (SAH), SHAP clarifies the influence of many input features—such as imaging biomarkers, clinical factors, or laboratory results—on model predictions. SHAP assigns Shapley values to each feature, quantifying its marginal contribution to a specific prediction and providing insights into the most relevant aspects for identifying SAH situations.

**Fig. 5:**
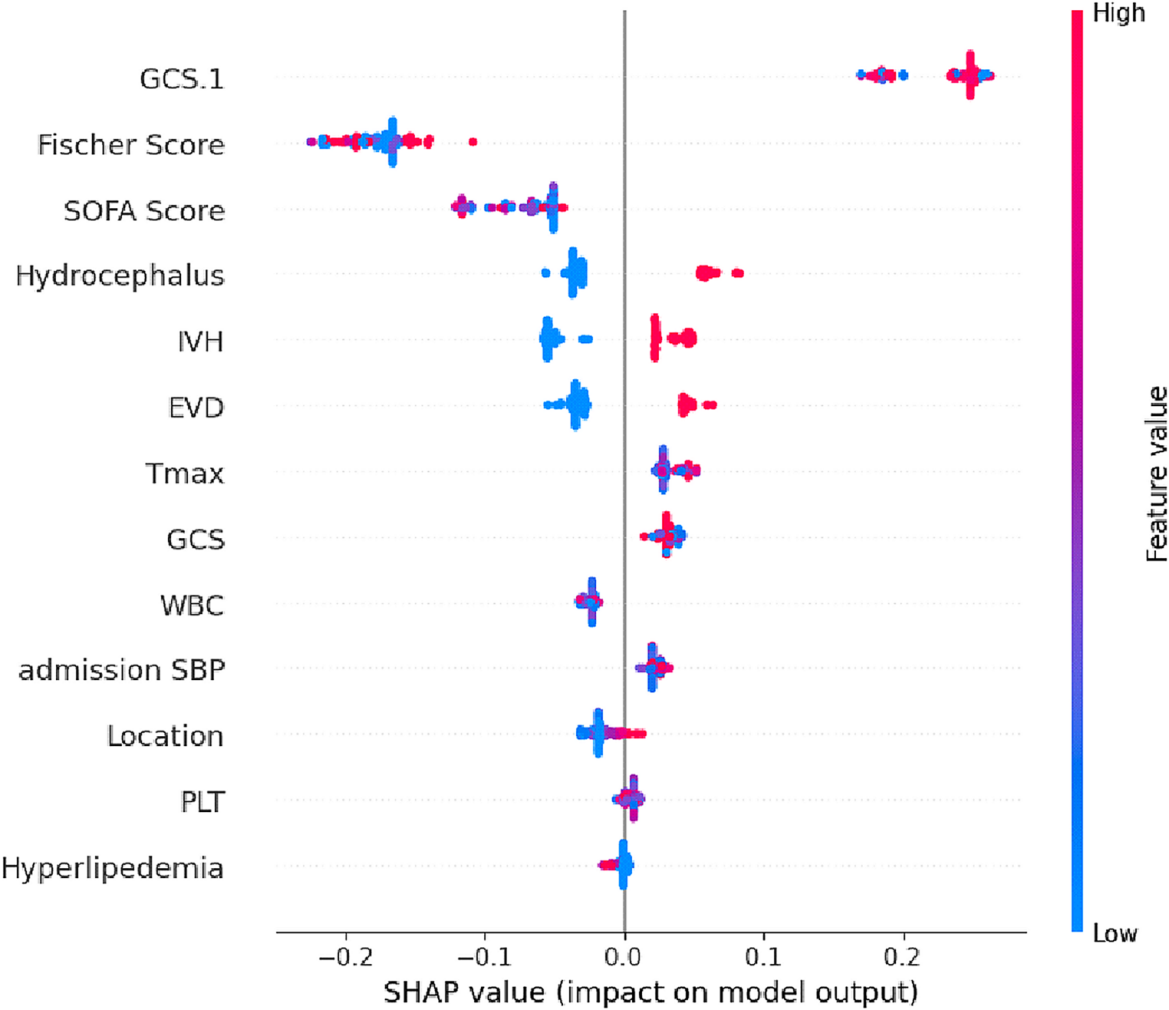
SHAP summary plot illustrating the impact of the top features on the model’s predictions

Figure 5 shows the SHAP summary plot of binary classification stage (1st stage) illustrating the impact of the top features on the model’s predictions for SAH classification. The x-axis denotes the SHAP values, reflecting the magnitude and direction of each feature’s impact on the model output. Features are prioritized based on their importance, with “GCS.1,” “Fisher Score,” and “SOFA Score” identified as the most influential. The color gradient, transitioning from blue (indicating low feature values) to red (indicating high feature values), emphasizes the distribution of feature values among individual forecasts. For instance, larger values of “GCS.1” (red) positively influence the model’s predictions, whereas lower values (blue) have the opposite effect. Likewise, characteristics like as “Hydrocephalus” and “IVH” exhibit significant contributions, indicating their clinical importance in the identification of SAH. This graphic highlight the essential impact of these features in influencing the model’s conclusions, offering a clear and interpretable account of feature significance in SAH diagnosis.

## Discussion on limitations and future directions

Our work presents a novel SAH dataset, providing a useful basis for severity prediction modeling, using the MRS as the ground truth. Our investigation revealed difficulties in forecasting results for Class 5 (MRS 5) and Class 6 (MRS 6), the most severe classes. Due to the severe nature of these cases and their limited representation, model performance was greatly impacted by prioritizing more frequent outcomes over rare, important ones.

We created a multi-phase strategy to better manage data imbalance in order to overcome these issues. With this method, instances are gradually categorized in phases: first, more general severity levels are grouped, and then, within each severity category, more precise predictions are made. This technique lessens the detrimental impacts of class imbalance on the model’s performance by first generating a generic classification and then zooming into particular classes. The first phase’s binary classification accuracy was over 90%. The “Good Outcome Class” (classes 0–3) and the “Poor Outcome Class” (classes 4–6) had 88% and 86% accuracy, respectively. This multi-phase approach demonstrated its efficacy in managing unbalanced data and forecasting severe SAH outcomes by greatly increasing model accuracy.

To improve the model’s generalizability, we used relevance-based feature ranking. This approach reduces the possibility of overfitting by choosing important features, which improves the model’s ability to generalize. We prioritized features using the Random Forest feature ranking technique, which also made the model simpler by concentrating on the factors that had the biggest effects.

Our findings have significant implications for clinical practice. With the use of our multi-phase approach, clinicians can classify patients according to their risk levels by using an organized, data-driven tool for determining the severity of SAH. By ensuring that patients receive therapies that are appropriate for their severity level, this stratification may assist optimize the allocation of resources. Furthermore, the predictive ability of the model across different severity levels supports data-driven decision-making by giving medical professionals vital information about the anticipated outcomes of their patients [58].

It is crucial to address the intrinsic data imbalance in SAH prediction models because it makes it difficult to predict uncommon but serious events with accuracy. Our results highlight the need for specialized methods to address data imbalance in order to enhance model performance at all severity levels.

Although our study offers insightful information, it also recognizes some limits. Model accuracy may have been impacted by the uneven severity class distribution, particularly when it comes to predicting minority classes. Furthermore, the accuracy of our forecasts may be impacted by problems with the data, such as missing numbers. Another issue is the model’s generalizability, since the dataset might not accurately reflect the variety of SAH patients, which would restrict its use in more extensive clinical contexts. Furthermore, even when class imbalance was addressed by methods like SMOTE, the model’s performance may be impacted since the synthetic samples produced by SMOTE might not always accurately reflect instances of the minority classes.

SMOTE has a number of drawbacks despite being an effective method for resolving data imbalance. Its generation of synthetic samples through interpolation between existing members of the minority class is a major problem. This procedure could produce fictitious, unrealistic samples that don’t fairly depict the data’s actual distribution. These artificial examples could add noise to the model, particularly if the minority class is underrepresented and has insufficient diversity. Additionally, SMOTE ignores possible class overlap, which may result in overfitting or incorrect classification, especially when used on high-dimensional data. Notwithstanding these limitations, the method can be helpful in distributing datasets for model training; nevertheless, its limitations should be carefully considered, and it might be advantageous to combine it with other tactics to enhance model performance and prevent the introduction of artificial sample bias.

In the future, a number of possible paths could enhance the prediction of SAH severity even more. Adding longitudinal and multimodal imaging data to our dataset would increase its feature space and provide us with a more comprehensive picture of how SAH develops. Accuracy could be increased across all severity groups using sophisticated balancing strategies like oversampling or the creation of synthetic data. Improved feature engineering may highlight minute patterns, bolstering the model’s capacity for prediction. Furthermore, in order to promote clinician trust and facilitate seamless integration into clinical workflows, it is imperative to increase model clarity [59].

The importance of predictive modeling for SAH severity in clinical practice is evaluated in this study, along with the advantages and drawbacks of our results. We emphasize the potential of machine learning to improve clinical decision-making and stress the significance of correcting data imbalances to increase accuracy across all SAH classes. By classifying patients according to the severity of SAH, our model suggests a multi-phase prediction technique that enables focused resource allocation and individualized care. We propose a number of future enhancements while recognizing several drawbacks, including an unbalanced dataset, possible biases brought on by poor data quality, limited generalizability, and the difficulty of understanding machine learning results in clinical contexts. These include adding a variety of clinical data to the dataset, using sophisticated resampling methods, honing feature engineering, enhancing model transparency, and carrying out thorough external validations. Ultimately, to optimize the model’s influence on SAH patient outcomes, successful clinical practice adoption necessitates teamwork.

## Conclusion

This study focuses on accurately predicting the severity of SAH to guide clinical decisions and improve patient outcomes. Using a three-stage classification framework with the MRS, we categorized SAH severity into “Good Outcome” (MRS levels 0, 1, 2, 3) and “Poor Outcome” (MRS levels 4, 5, 6). Feature selection was conducted using a Random Forest algorithm to identify the top 20 features for SAH severity prediction. Thirteen machine learning models were evaluated at each stage, with the best-performing classifiers selected to optimize results. The results showed that the multi-stage approach improved accuracy across unbalanced classes, with the binary classification achieving 90% accuracy using Extra Trees. In the second stage, the Random Forest model reached 88% accuracy for the “Good Outcome” group, and 86% for the “Poor Outcome” group in the third stage. These findings highlight the effectiveness of combining established and innovative techniques for developing clinically useful SAH prediction models. This work offers insights for academics, clinicians, and policymakers, emphasizing the need for further collaboration and practical implementation to enhance patient care and outcomes.

## Electronic supplementary material

Below is the link to the electronic supplementary material.

Supplementary Material 1

## Data Availability

The preprocessed version of the dataset can be made available upon reasonable request to the corresponding author.
