# Supplementary material for: Multi-class subarachnoid hemorrhage severity prediction: addressing challenges in predicting rare outcomes
Source: Neurosurg Rev. 2025 Jul 10;48(1):554. doi: 10.1007/s10143-025-03678-9 (PMC12246023; doi:10.1007/s10143-025-03678-9)
Supplement: Supplementary file 1 — Supplementary Material 1 [file 10143_2025_3678_MOESM1_ESM.docx]

**Supplementary Materials**

**Supplementary Table S1.** Statistical analysis of the prime characteristic of the subjects’ data

| **Item** | **Survived** | **Death** | **Total** | **Method** | **Statistic** | ***P* value** |
| --- | --- | --- | --- | --- | --- | --- |
| **Gender** |  |  |  | Chi-square test | X^2^ = 0.2412 | 0.6233 |
| • Male (%) | 350 (67.3%) | 11 (73.3%) | 361 (67.5%) |  |  |  |
| • Female (%) | 170 (32.7%) | 4 (26.7%) | 174 (32.5%) |  |  |  |
| **Age** |  |  |  | Rank-sum test | Z = 6.42 | < 0.00000001 |
| • *N* (missing) | 520 (0) | 15 (0) | 535 (0) |  |  |  |
| • Mean ± SD | 45.64 ± 11.87 | 42.36 ± 12.27 | 45.67 ± 11.84 |  |  |  |
| • Median | 46 | 41 | 46 |  |  |  |
| • Q1, Q3 | 38, 53 | 36, 48 | 38, 53 |  |  |  |
| • Min, max | 15, 99 | 24, 72 | 15, 99 |  |  |  |
| **Fischer Score** |  |  |  | Rank-sum test | Z = 6.40 | < 0.00000001 |
| • *N* (missing) | 520 (0) | 15 (0) | 535 (0) |  |  |  |
| • Mean ± SD | 2.31 ± 1.33 | 3.93 ± 0.46 | 2.35 ± 1.33 |  |  |  |
| • Median | 2 | 4 | 2 |  |  |  |
| • Q1, Q3 | 1, 4 | 4, 4 | 1, 4 |  |  |  |
| • Min, max | 0, 5 | 3, 5 | 0, 5 |  |  |  |
| **SOFA Score** |  |  |  | Rank-sum test | Z = 6.40 | < 0.000000001 |
| • *N* (missing) | 519 (1) | 15 (0) | 534 (1) |  |  |  |
| • Mean ± SD | 5.08 ± 3.28 | 9.07 ± 3.73 | 5.18 ± 3.36 |  |  |  |
| • Median | 4 | 10 | 4 |  |  |  |
| • Q1, Q3 | 2, 8 | 8, 11.5 | 2, 8 |  |  |  |
| • Min, max | 0, 16 | 0, 13 | 0, 16 |  |  |  |
| **H&H Score** |  |  |  | Rank-sum test | Z = 6.40 | < 0.000000001 |
| • *N*(missing) | 520 (0) | 15 (0) | 535 (0) |  |  |  |
| • Mean ± SD | 1.88 ± 1.46 | 4.87 ± 0.35 | 1.96 ± 1.53 |  |  |  |
| • Median | 1 | 5 | 1 |  |  |  |
| • Q1, Q3 | 1, 3 | 5, 5 | 1, 3 |  |  |  |
| • Min, max | 0, 9 | 4, 5 | 0, 9 |  |  |  |
| **GCS** |  |  |  | Rank-sum test | Z = 6.40 | < 0.000000001 |
| • *N* (missing) | 520 (0) | 15 (0) | 535 (0) |  |  |  |
| • Mean ± SD | 12.21 ± 3.34 | 4 ± 1.13 | 11.86 ± 3.59 |  |  |  |
| • Median | 14 | 3.5 | 14 |  |  |  |
| • Q1, Q3 | 37, 62 | 3, 5 | 9.5, 14 |  |  |  |
| • Min, max | 12, 14 | 3, 6 | 12, 14 |  |  |  |
| **Tmax** |  |  |  | Rank-sum test | Z = 6.34 | < 0.000000002 |
| • *N* (missing) | 424 (96) | 15 (0) | 439 (96) |  |  |  |
| • Mean ± SD | 37.39 ± 2.14 | 37.62 ± 1.53 | 37.39 ± 2.12 |  |  |  |
| • Median | 37.1 | 38.4 | 37.1 |  |  |  |
| • Q1, Q3 | 37, 38.43 | 35, 95, 38, 85 | 37, 38.5 |  |  |  |
| • Min, max | 0, 40.1 | 35, 39.2 | 0, 40.1 |  |  |  |
| **ECG** |  |  |  | Rank-sum test | Z = 6.40 | < 0.000000001 |
| • *N* (missing) | 520 (0) | 15 (0) | 535 (0) |  |  |  |
| • Mean ± SD | 1.69 ± 2.5 | 4.8± 2.27 | 1.78 ± 2.55 |  |  |  |
| • Median | 0 | 4 | 1 |  |  |  |
| • Q1, Q3 | 56.5, 75.4 | 4, 4.5 | 0, 3 |  |  |  |
| • Min, max | 0, 3 | 2, 9 | 0, 9 |  |  |  |
| **PLT** |  |  |  | Rank-sum test | Z = 6.40 | < 0.000000001 |
| • *N*(missing) | 519 (1) | 15 (0) | 534 (1) |  |  |  |
| • Mean ± SD | 282.39± 74.40 | 236.30 ± 82.31 | 279.96 ± 75.77 |  |  |  |
| • Median | 284.5 | 247 | 281.5 |  |  |  |
| • Q1, Q3 | 233.75, 333 | 145, 300 | 229, 333 |  |  |  |
| • Min, max | 23, 536 | 109, 372 | 23, 536 |  |  |  |
| **WBC** |  |  |  | Rank-sum test | Z = 6.40 | < 0.000000001 |
| • *N* (missing) | 520 (0) | 15 (0) | 535 (0) |  |  |  |
| • Mean ± SD | 13.34 ± 5.01 | 17.64 ± 3.58 | 13.43 ± 4.99 |  |  |  |
| • Median | 12.5 | 18.3 | 12.7 |  |  |  |
| • Q1, Q3 | 9.7, 16.2 | 16.3, 19.2 | 9.8, 16.28 |  |  |  |
| • Min, max | 0, 35 | 11.6, 23.7 | 0, 35 |  |  |  |
| **Admision SBP** |  |  |  | Rank-sum test | Z = 6.40 | < 0.000000001 |
| • *N* (missing) | 518 (2) | 15 (0) | 533 (2) |  |  |  |
| • Mean ± SD | 172 ± 34.57 | 152.6 ± 47.87 | 171.46 ± 35.09 |  |  |  |
| • Median | 173 | 160 | 172 |  |  |  |
| • Q1, Q3 | 203, 312 | 140, 200 | 140, 200 |  |  |  |
| • Min, max | 70, 300 | 70, 220 | 70, 300 |  |  |  |
| **Number of Aneurysm** |  |  |  | Rank-sum test | Z = 6.40 | < 0.000000001 |
| • *N* (missing) | 520 (0) | 15 (0) | 535 (0) |  |  |  |
| • Mean ± SD | 0.93 ± 0.92 | 1.4 ± 1.12 | 0.94 ± 0.92 |  |  |  |
| • Median | 1 | 1 | 1 |  |  |  |
| • Q1, Q3 | 1, 1 | 1, 1 | 1, 1 |  |  |  |
| • Min, max | 0, 11 | 1, 5 | 0, 11 |  |  |  |
| **Outcome (%)** | 520 (97.20) | 15 (2.80) | 535 |  |  |  |


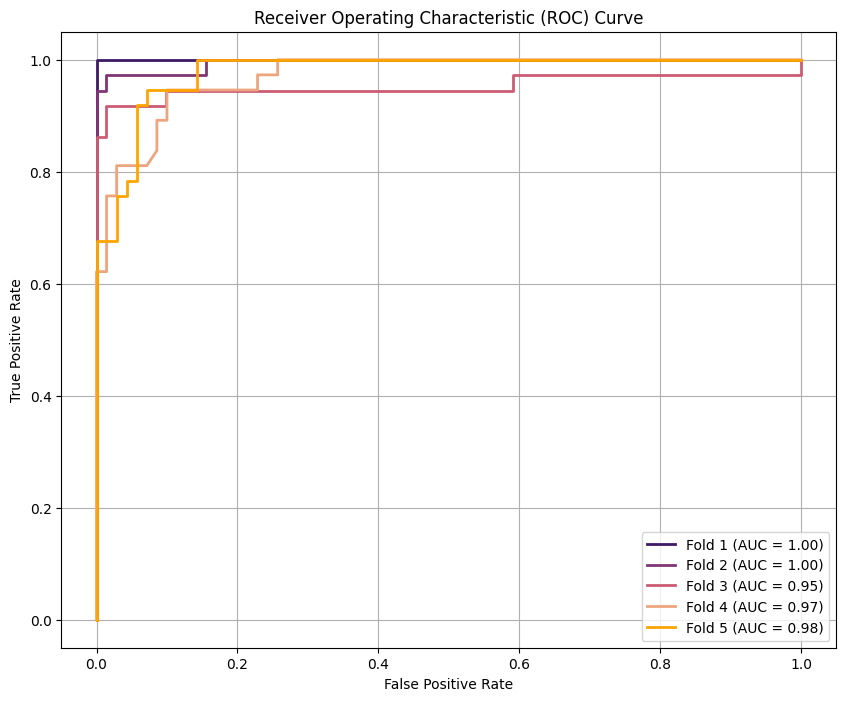


**Supplementary Figure S1.** AUC curve for the ExtraTreesClassifier in first stage classification.


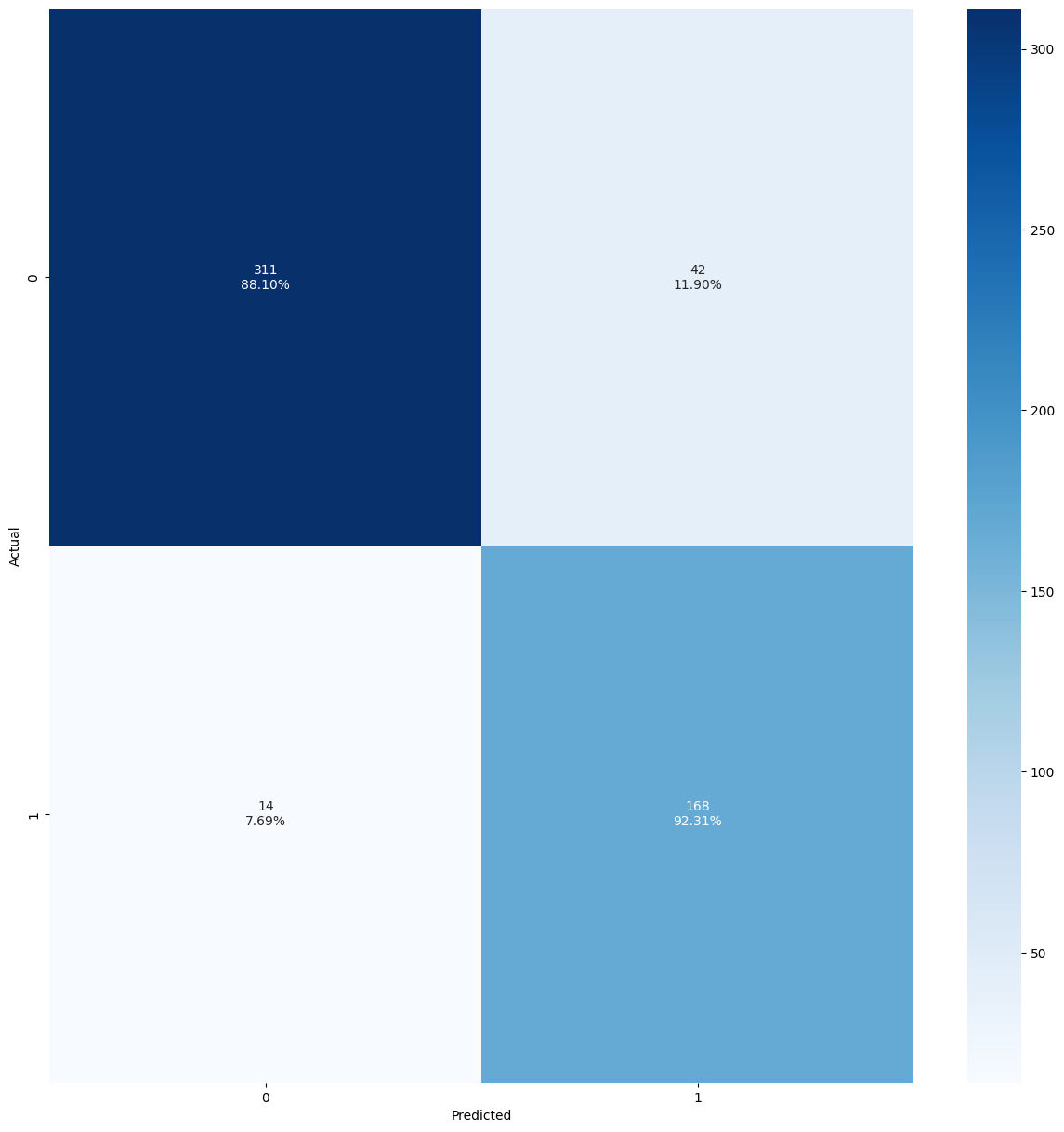


**Supplementary Figure S2.** Confusion Matrix for the ExtraTreesClassifier in the first stage of classification.


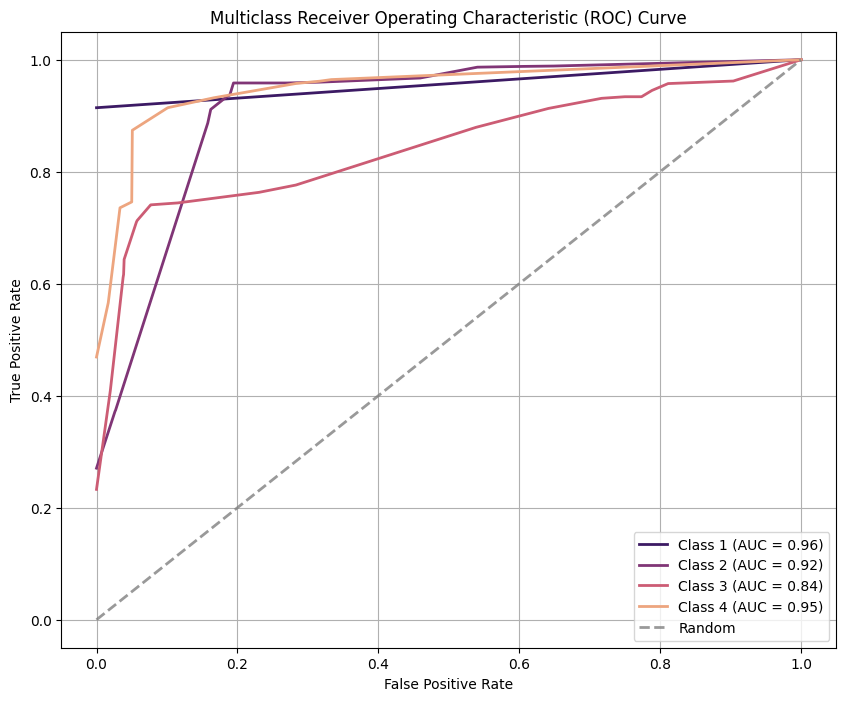


**Supplementary Figure S3.** AUC curve for the Random Forest Classifier in the second stage of classification.


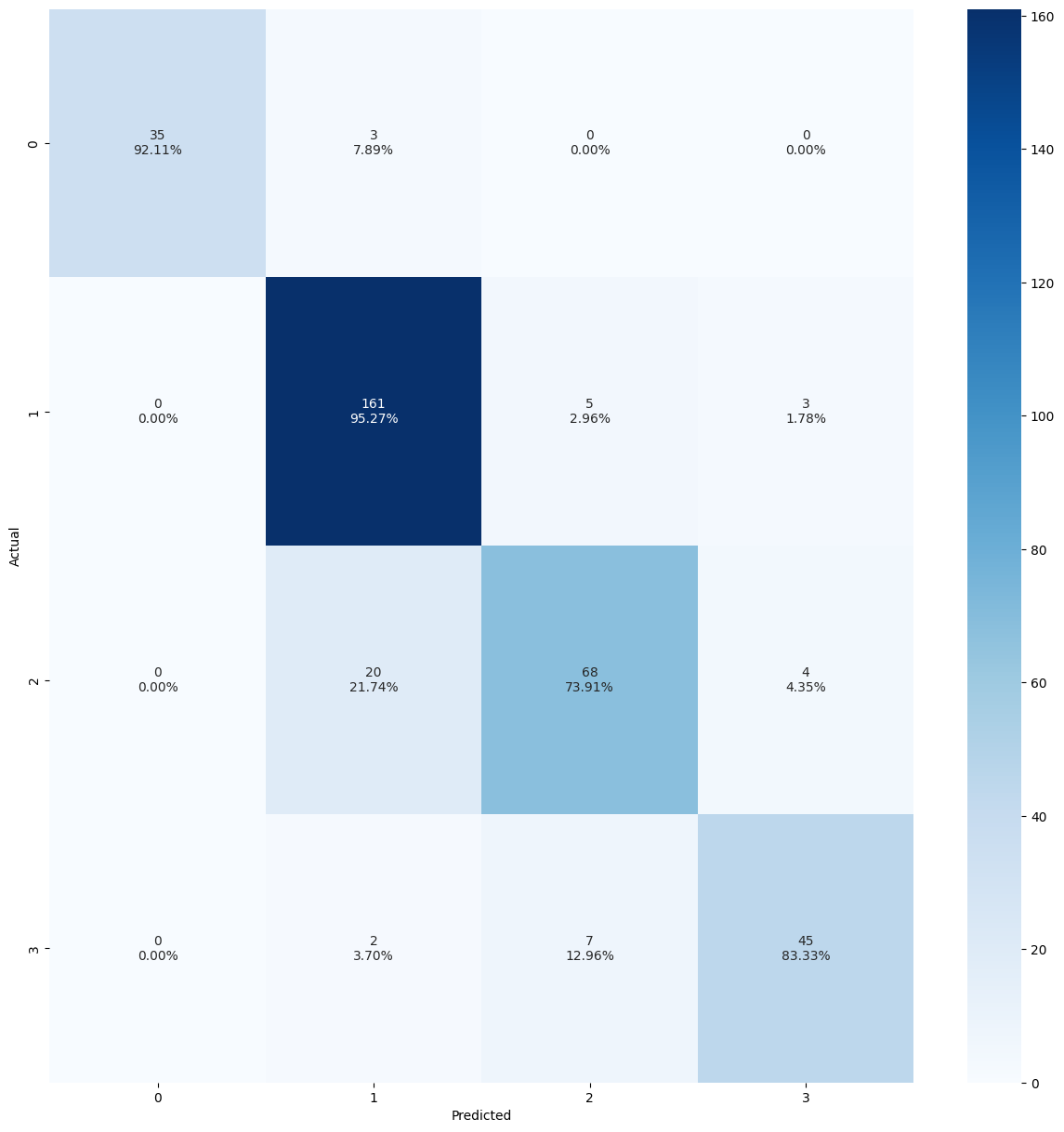


**Supplementary Figure S4.** Confusion Matrix for the Random Forest Classifier in the second stage of classification.


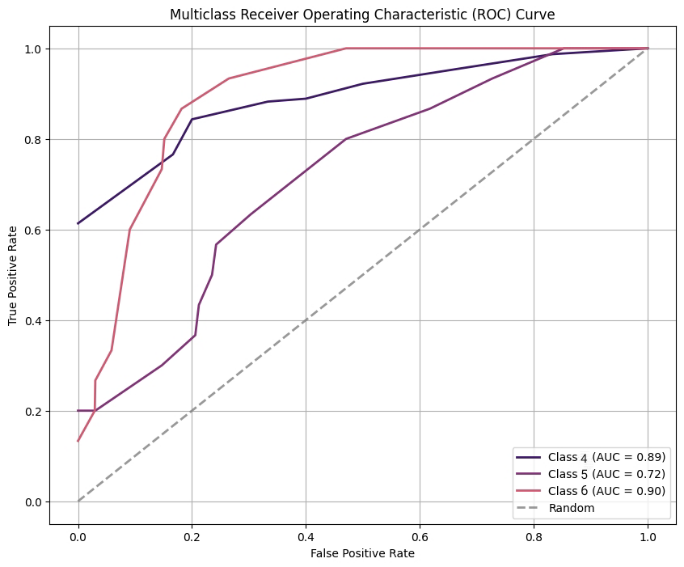


**Supplementary Figure S5.** AUC curve for the Random Forest Classifier in the third stage of classification.


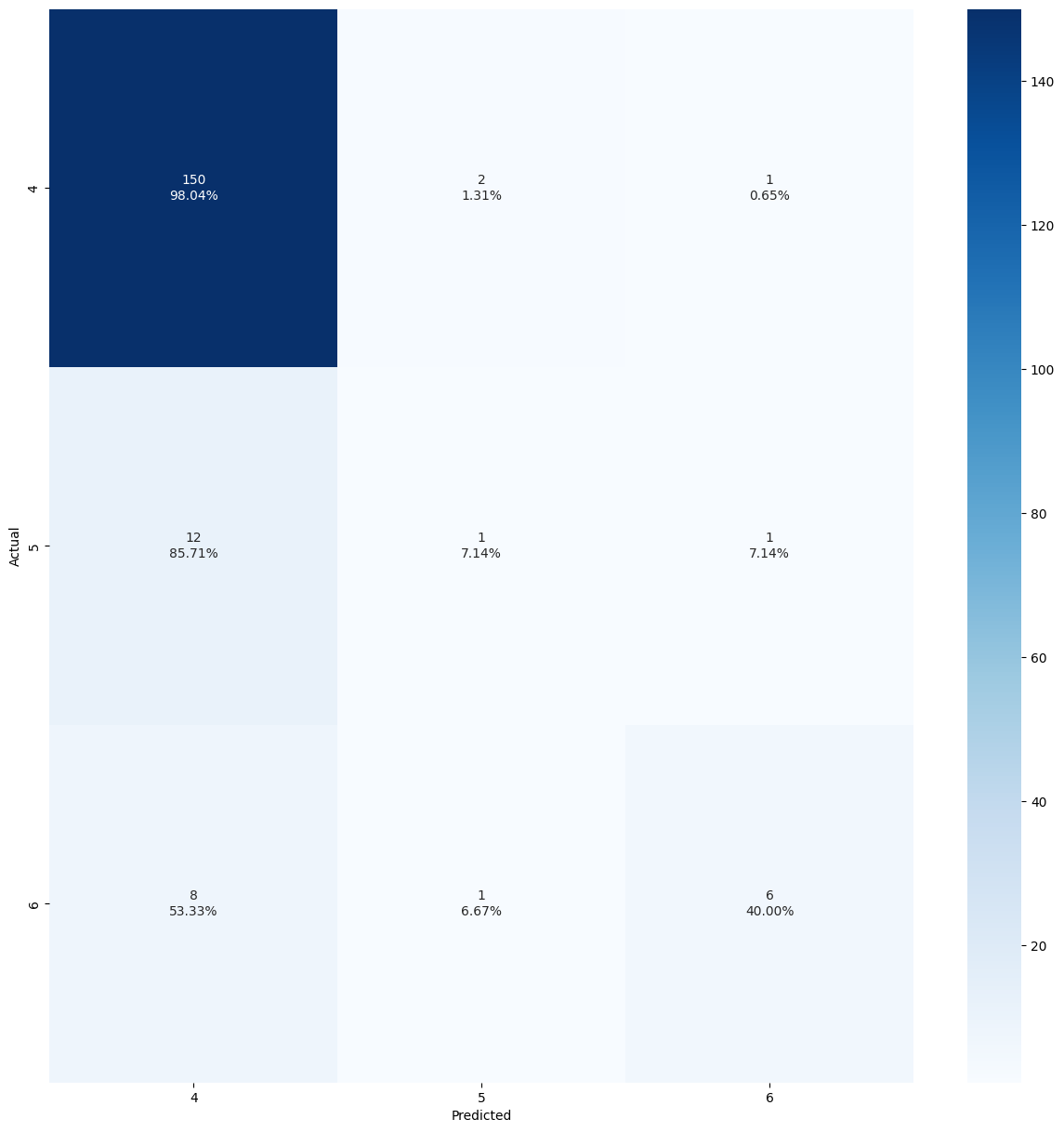


**Supplementary Figure S6.** Confusion Matrix for the Random Forest Classifier in the third stage of classification.
